# The Nematic Phases of Bent-Core Liquid Crystals

**DOI:** 10.1002/cphc.201400014

**Published:** 2014-04-02

**Authors:** Helen F Gleeson, Sarabjot Kaur, Verena Görtz, Abdel Belaissaoui, Stephen Cowling, John W Goodby

**Affiliations:** [b] Department of Chemistry University of York, Heslington, York YO10 5DD (UK); [c] Department of Chemistry Lancaster University, Lancaster LA1 4YB (UK)

**Keywords:** bent-core molecules, liquid crystals, nematic phases, oxadiazoles, physical properties

## Abstract

Over the last ten years, the nematic phases of liquid crystals formed from bent-core structures have provoked considerable research because of their remarkable properties. This Minireview summarises some recent measurements of the physical properties of these systems, as well as describing some new data. We concentrate on oxadiazole-based materials as exemplars of this class of nematogens, but also describe some other bent-core systems. The influence of molecular structure on the stability of the nematic phase is described, together with progress in reducing the nematic transition temperatures by modifications to the molecular structure. The physical properties of bent-core nematic materials have proven difficult to study, but patterns are emerging regarding their optical and dielectric properties. Recent breakthroughs in understanding the elastic and flexoelectric behaviour are summarised. Finally, some exemplars of unusual electric field behaviour are described.

## 1. Introduction

The nematic phases formed by bent-core liquid crystals were propelled into the spotlight about ten years ago with the first experimental observations that could have indicated the highly sought-after thermotropic biaxial nematic phase.^1^, ^2^ The possible manifestation of biaxiality in such nematic materials was deduced through techniques including polarising microscopy, conoscopy, deuterium NMR spectroscopy,^3^ X-ray scattering, infrared spectroscopy,^4^ light scattering,^5^ ^13^C NMR spectroscopy^6^ and Raman spectroscopy.^7^ All of these approaches have their advantages and complications, for example, several rely on large external fields, which can influence the nematic structure, are restricted in the geometry they can examine, or rely on sophisticated models for the interpretation of the measurements. In fact, over the past ten years it has been suggested that in many (but not all) cases, the unusual behaviour that can be observed in the nematic phases formed from bent-core materials is the result of a high propensity for the formation of cybotactic smectic-C (SmC) clusters.^8^ In some cases, the association is only one or two molecules, and transitory biaxial order can be induced.^9^ These fascinating and complex materials have stimulated considerable research, and an excellent review was written by Tschierske and Photinos in 2010.^10^

Measurement of the physical properties of the nematic phases formed by bent-core molecules has proven rather challenging because of the high temperatures at which the nematic phase often occurs (>150 °C) and because it has been difficult to obtain the high-quality monodomain alignment necessary for robust measurements. Nonetheless, reports of the elastic constants, dielectric behaviour, flexoelectric coefficients and unusual electro-optic behaviour are growing. Herein, we review the physical properties of nematic phases formed by bent-core liquid crystals. The oxadiazole-based materials are used as exemplars, though some other systems are also described. The paper is organised as follows. Section 2 describes the influence of molecular structure on the nematic-phase range in bent-core liquid crystals. As has already been mentioned, a key issue has been to reduce the temperatures at which the nematic phase is exhibited and we describe structural modifications that result in significantly lower temperature nematic phases. Section 3 describes the optical properties, dielectric behaviour, elasticity and flexoelectricity in bent-core nematic liquid crystals. Finally, Section 4 considers some of the unusual electro-optic behaviour reported for these compounds.

## 2. Relationship between Structure and Nematic-Phase Range

The formation of nematic phases in bent-core liquid-crystalline materials relies on a subtle balance between order and disorder. Although there are well-established design rules for calamitic nematic materials, it has proven rather more difficult to synthesise bent-core molecules with nematic phases, and especially to control the extent and absolute temperature of the nematic regime. This section describes the influence of specific design variations allowing some general conclusions to be drawn. However, it should be emphasised that many of the molecules based on the oxadiazole core simply do not exhibit nematic phases, which shows that the subtle balance of order and disorder is very hard to achieve in bent-core compounds.

### 2.1. High-Temperature Oxadiazole-Based Compounds

#### 2.1.1. Oxadiazole-Based Materials with Terminal Alkyl Chains

**Figure 1:**
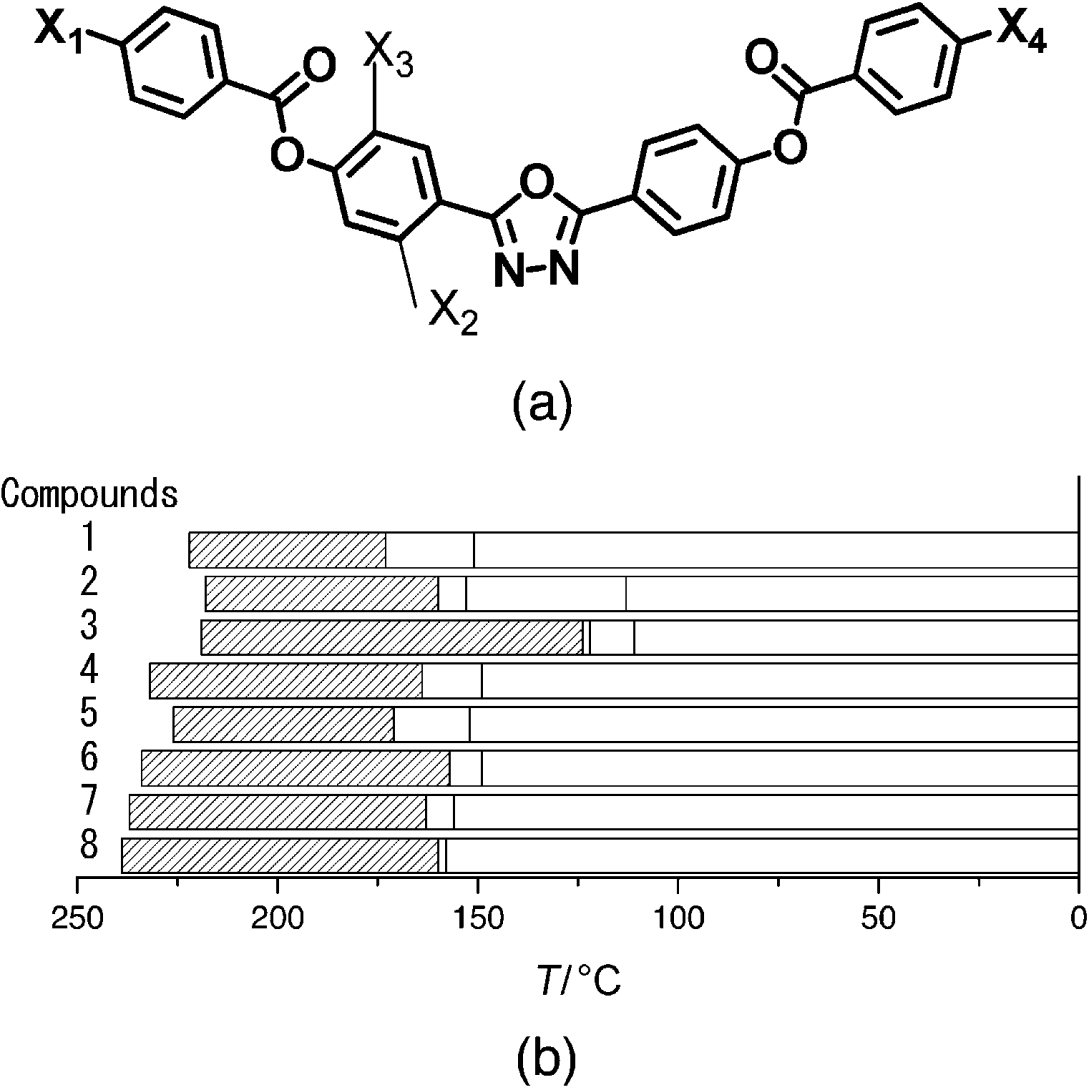
a) General structure of the oxadiazole-based liquid-crystalline materials referred to in Table 1. b) The nematic-phase range of the compounds obtained by differential scanning calorimetry (10 °C min^−1^) on cooling. Table 1 gives details of the structures and the width of the nematic phase; the dense slant pattern in (b) depicts the extent of the nematic phase in all compounds.

The nematic phases of the very first oxadiazole-based compounds, reported because of their possible biaxiality, were at rather high temperatures, with most exhibiting the nematic phase at temperatures above 150 °C. The general structure of the materials is shown in Figure 1 a, with the phase behaviour shown schematically in Figure 1 b. Table 1 gives details of the terminal chains (X_1_ and X_4_) in Figure 1 a and substitution on the aromatic core (X_2_ and X_3_). Here, we do not consider the nature of the higher-order phases below the nematic phase, though such details are given elsewhere. In all of the Tables we list the published work relating to specific compounds possible; where no reference is listed, the data have not been published elsewhere.

**Table 1:** Influence of molecular structure on the nematic-phase range for the oxadiazole compounds illustrated in Figure 1, in which the terminal groups and lateral substituents are varied.

Compound	X_1_	X_2_	X_3_	X_4_	Nematic range [°C] (width [K])	Refs.
**1**	C_7_H_15_	H	H	C_7_H_15_	173–222 (49)	1, 2, 11 12, 13, 14
**2**	C_7_H_15_	F	H	C_7_H_15_	160–218 (58)	13
**3**	C_7_H_15_	F	F	C_7_H_15_	124–219 (95)	13
**4**	C_7_H_15_	H	H	C_6_H_13_	171–226 (55)	13
**5**	C_7_H_13_	H	H	C_5_H_11_	164–232 (68)	13, 14
**6**	C_6_H_13_	H	H	C_5_H_11_	163–237 (74)	13
**7**	C_7_H_15_	H	H	C_4_H_9_	157–234 (77)	13
**8**	C_6_H_13_	H	H	C_4_H_9_	160–239 (79)	13

All of compounds **1**–**8** exhibit wide, high-temperature nematic phases. Compounds **1**–**3** have identical terminal chains (X_1_ and X_4_), but differ in the fluoro substituents on the inner phenylene group. It is apparent that a single fluorine group at the X_2_ position slightly increases the nematic-phase range as well as causing a small reduction in the transition temperatures. However, fluoro substitution in both positions has a remarkable effect, almost doubling the nematic-phase range achieved by reducing the higher order to nematic phase transition temperature.

Compounds **1** and **4**–**8** show the effect of decreasing the length of the terminal alkyl chains (X_1_ and X_4_) on the width of the nematic phase. The overall trend is that the phase range increases as the total chain length reduces. Similar behaviour is observed in calamitic materials, in which a smaller chain length tends to favour the nematic phase over a higher-order smectic phase. In general, an overall reduction in the length of the terminal chains tends to both reduce the temperature of the liquid crystal to nematic phase transition and to increase the clearing point.

#### 2.1.2. Oxadiazole-Based Materials with Terminal Alkoxy Chains

**Figure 2:**
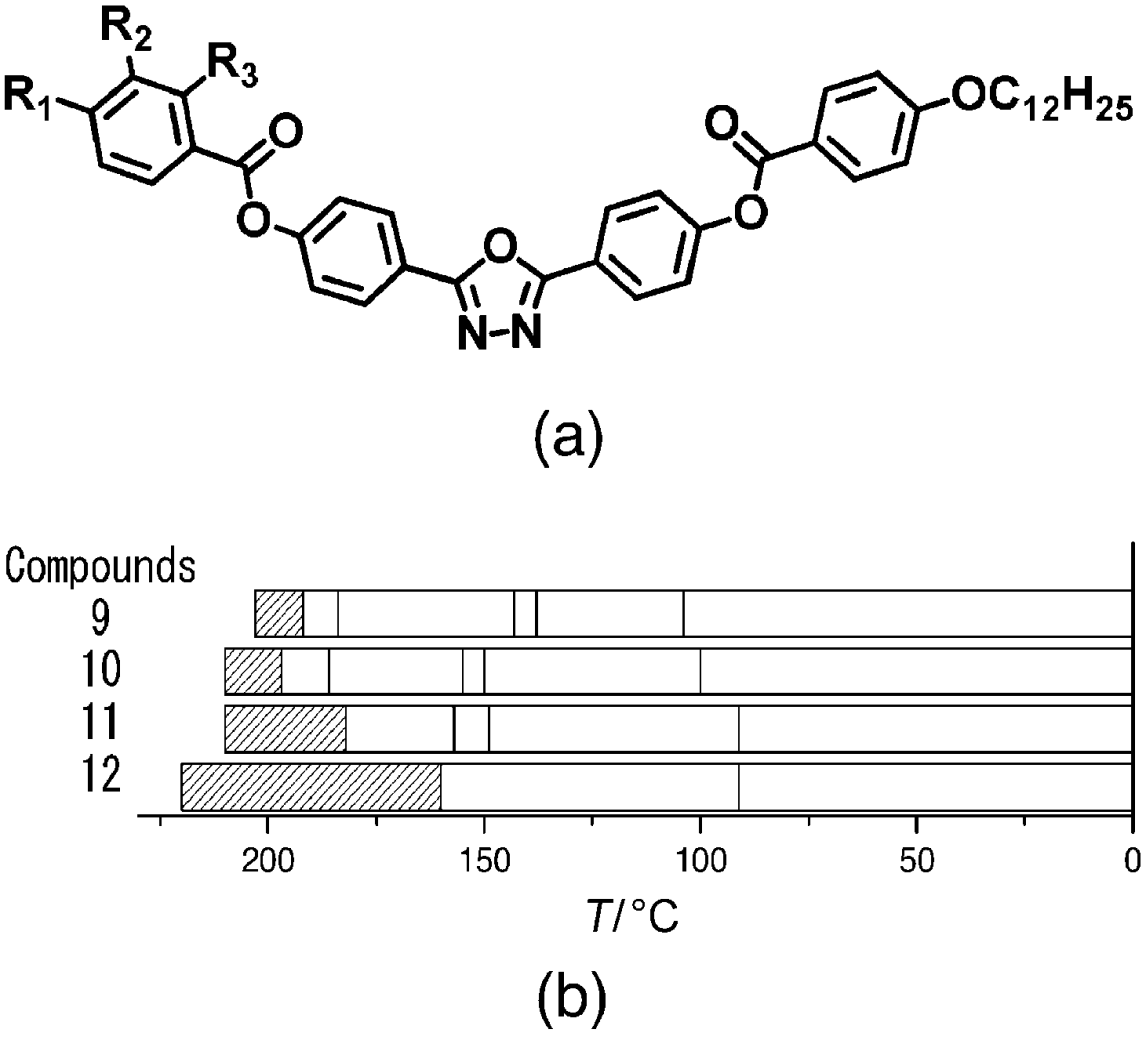
a) General structure of the oxadiazole-based liquid-crystalline materials referred to in Table 2. b) The nematic-phase range of the compounds obtained by differential scanning calorimetry (10 °C min^−1^) on cooling. Table 2 gives details of the structures and the width of the nematic phase; the dense slant pattern in (b) depicts the extent of the nematic phase in all compounds.

Many of the earliest bent-core nematogens included a dodecyloxy terminal chain, such as is shown in Figure 2 a. Again, some general trends are clear. Longer chain lengths are clearly associated with a reduced nematic-phase range; compound **9**, which has two dodecyloxy chains, has a phase range of only 11 K (see Table 2). The phase range is extended to 60 K when a nonyloxy chain is introduced on one side (compound **12**). Again, fluoro substitution (compound **10**) has a significant influence on the phase range, though in this case the outermost aromatic group is fluorinated and the substitution serves to severely reduce the stability of the nematic phase. Compound **10** has a nematic phase that is less than half as wide as that of compound **11** with the decrease in nematic range in compound **10** resulting from increased stability of the higher-ordered liquid-crystalline phases. Clearly the effect of substituting fluorine atoms onto the molecular core is very sensitive to the position of the substitution. In compound **10**, the substitution is likely to influence both the molecular dipole and the conformers that can be adopted and the outcome is to reduce the nematic stability. Finally for this group of materials, a dramatic increase in the nematic phase stability is again seen when the chain length at R_1_ is reduced further, for example in compound **12** in which R_1_ is a pentyl chain. Although not as wide as compounds **1**–**8**, which all included relatively short alkyl terminal chains, the nematic phase regime in compound **12** is 60 K wide, which indicates significant stability of the phase.

**Table 2:** Influence of molecular structure on the nematic-phase range for oxadiazole compounds, in which only one terminal chain is varied. The influence of fluorine substitution on the outer phenyl ring is compared in compounds 10 and 11.

Compound	R_1_	R_2_	R_3_	Nematic range [°C] (width [K])	Refs.
**9**	C_12_H_25_O	H	H	192–203 (11)	1, 2, 11, 12, 15
**10**	C_9_H_19_O	F	F	197–210 (13)	12, 15
**11**	C_9_H_19_O	H	H	182–210 (28)	12, 15
**12**	C_5_H_11_	H	H	155–215 (60)	7, 12, 13, 14, 15, 16, 17

Zafiropoulos et al.^18^ made a dramatic structural variation to the oxadiazole core, by including only one terminal alkoxy chain. A slight odd–even effect is reported for the series and the lowest-temperature nematic onset was for the octyloxy chain with a nematic onset at approximately 105 °C and a range of about 60 K.

### 2.2. Lateral Substitution of Oxadiazoles To Reduce Transition Temperatures

#### 2.2.1. Lateral Substitution on the Outer Phenyl Rings

**Figure 3:**
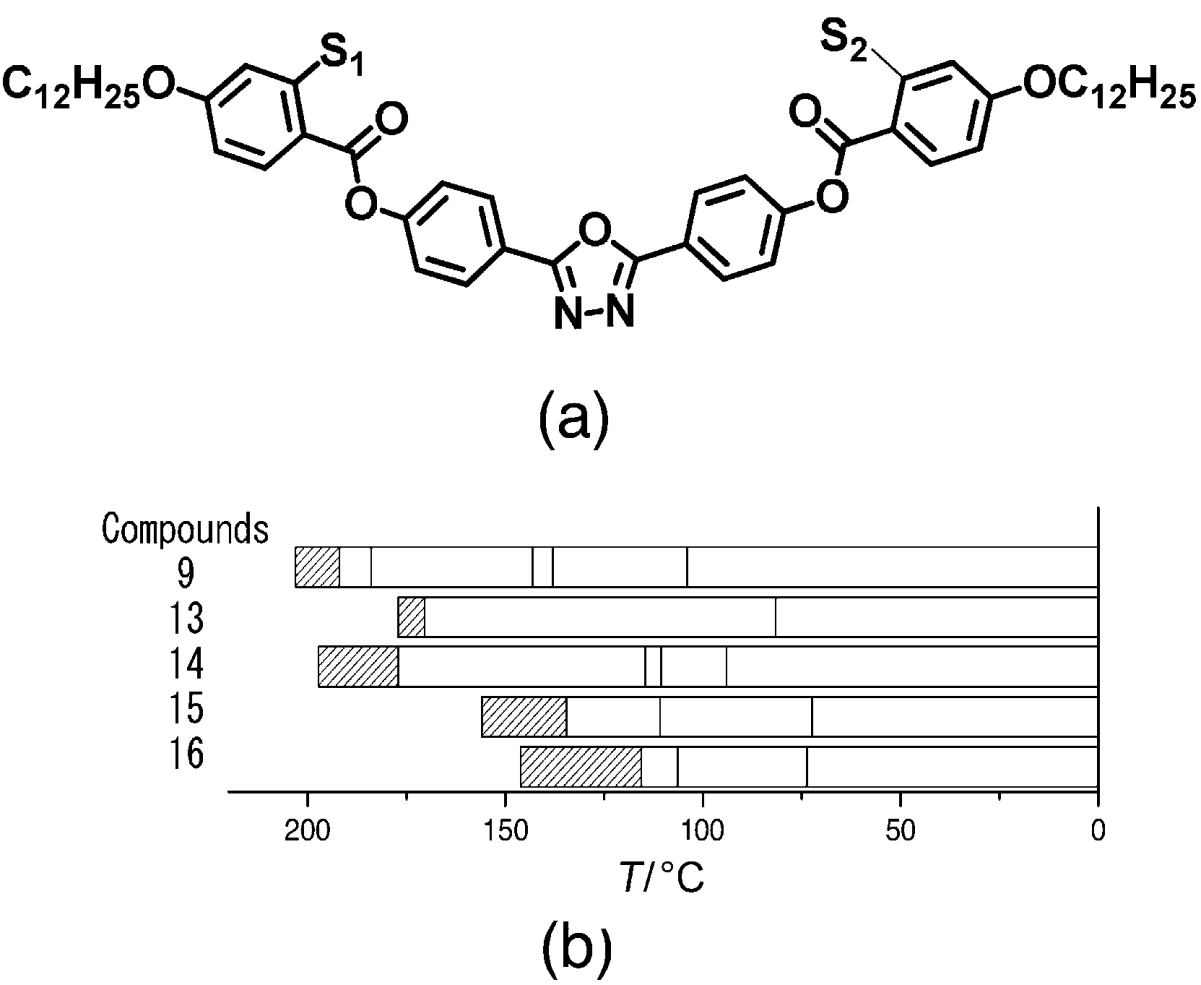
a) General structure of the laterally substituted oxadiazole-based materials referred to in Table 3. Note that in contrast to Figure 1 and Table 1, the terminal chains are alkoxy units for this group of compounds. b) The nematic-phase range of the compounds obtained by differential scanning calorimetry (10 °C min^−1^) on cooling. Table 3 gives details of the structures and the width of the nematic phase; the dense slant pattern in (b) depicts the extent of the nematic phase in all compounds.

A well-known strategy for reducing the phase transition temperatures in nematogens is to include lateral substituents, thereby reducing the molecular length/width ratio. Figure 3 and Table 3 show the outcome of a strategy to include both bulky and polar groups as lateral substituents.

**Table 3:** Influence of molecular structure on the nematic-phase range for oxadiazole compounds with variation in the lateral substituents.

Compound	S_1_	S_2_	Nematic range [°C] (width [K])	Refs.
**9**	H	H	192–203 (11)	1, 2, 11, 12, 15
**13**	OMe	OMe	88.8–95.4 (6.6)	–
**14**	F	F	177.1–197.3 (20.2)	–
**15**	Cl	Cl	155.9–134.6 (21.3)	–
**16**	OMe	F	115.5–146.1 (30.4)	–

For compounds **9** and **13**–**16** the nematic phases remain at high temperatures, well above 150 °C. The fluorine-substituted material, compound **14**, once again has a relatively wide nematic range, twice as wide as that of the parent compound (**9**). The methoxy substituent (compound **13**) clearly adversely affects the ability to form a liquid-crystal phase, with an extremely narrow nematic range. Substitution of chlorine has a similar effect on the phase range to fluorination (compare compounds **14** and **15**), but has the additional beneficial effect of substantially reducing the phase transition temperatures. Asymmetric substitution has the most beneficial effect, both broadening the phase range to 30 K and at the same time reducing the whole nematic phase regime to under 150 °C.

Samulski and co-workers^19^, ^20^ have substituted lateral methyl groups as an approach to reduce the nematic temperature range in oxadiazole-based materials. The most successful strategy was asymmetric substitution, by which it was possible to produce materials with a nematic onset temperature below 100 °C.

#### 2.2.2. Inclusion of Branches in the Terminal Chains

**Figure 4:**
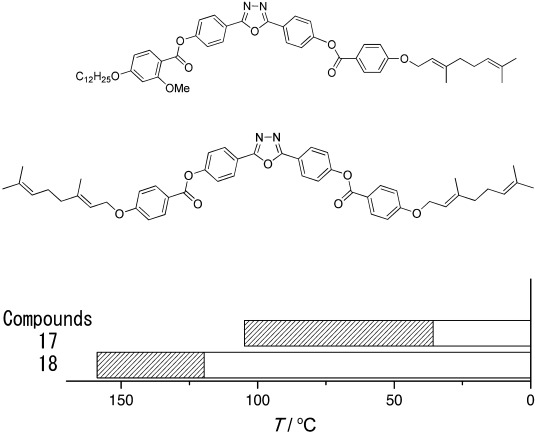
Structures of the oxadiazole-based liquid-crystalline materials with geranyloxy chains referred to in Table 4. b) The nematic-phase range of the compounds obtained by differential scanning calorimetry (10 °C min^−1^) on cooling. Table 4 gives details of the width of the nematic phase; the dense slant pattern in (b) depicts the extent of the nematic phase in all compounds.

An additional strategy that is well known for reducing liquid-crystal phase transition temperatures is the inclusion of branches on the terminal chains. Compounds **17** and **18** (Figure 4 and Table 4) are two examples of oxadiazole molecules with geranyloxy chains. Consider first compound **17**; asymmetric substitution onto the core was seen in Section 2.2.1 to have an extremely beneficial effect both on the nematic-phase range and on reducing the temperature at which the phase occurs. In compound **17**, the asymmetric core substitution is supplemented with a single geranyloxy chain, and the remarkable result is the oxadiazole-based material with a very low temperature nematic phase. The nematic phase is also wide (≈70 K), though the width reduces in compound **18** (40 K) when both terminal chains are geranyloxy based.

**Table 4:** Influence of molecular structure on the nematic-phase range for oxadiazole compounds with geranyloxy chains.

Compound	Nematic range [°C] (width [K])	Refs.
**17**	35.7–104.8 (69.1)	–
**18**	119.6–158.5 (39.2)	21

### 2.3. Nematic Ranges of Other Bent-Core Liquid Crystals

The oxadiazole materials are clearly important materials in studies of the nematic phases formed from bent-core molecules. However, there are many other bent-core nematogens, some of which are mentioned here to demonstrate the wide varieties of structure that have been synthesised. Table 5 shows the molecular structures of some of the materials that form especially low-temperature bent-core nematic phases, together with details of the transition temperatures and key references.

**Table 5:** Molecular structures and transition temperatures of some bent-core materials with low-temperature nematic phases.

Compound	Nematic range [K]	Refs.
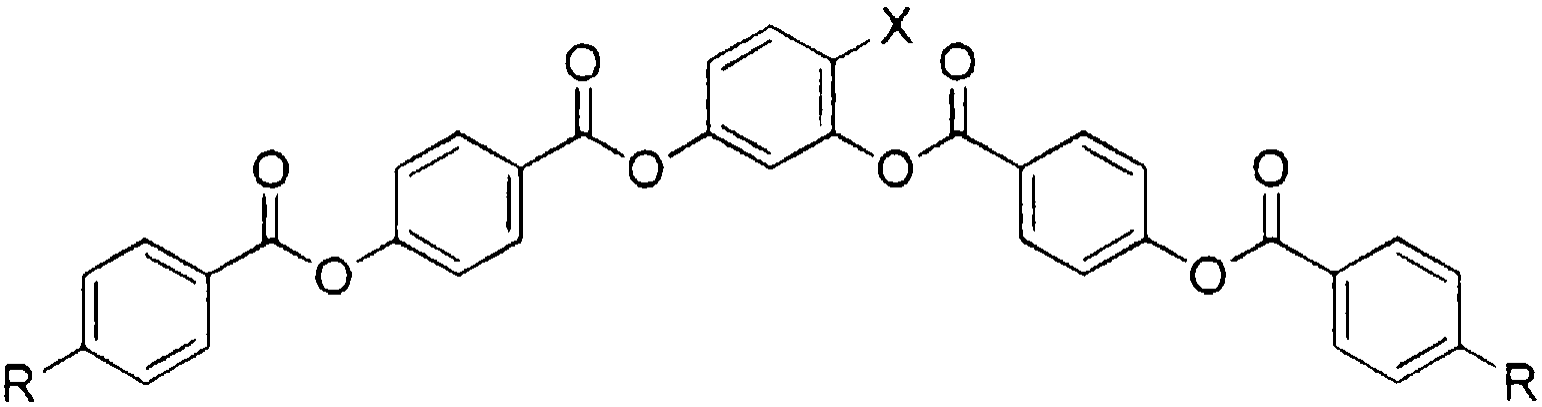	9	22
4-Chlororesorcinol bis[4-(4-*n*-alkyloxybenzoyloxy)benzoate] 86 (X 69) N 95 I R=OC_9_H_19_ and X=Cl gives the widest low-temperature nematic phase. Other substitution gives smaller nematic ranges or monotropic phases.			
		
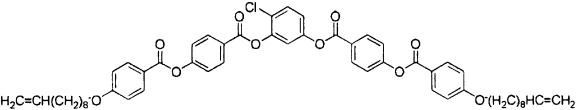	79	23
4-Chloro-1,3-phenylene bis-4-[4′-(9-decenyloxy) benzoyloxy]benzoate (ClPbis10BB) 70 N 78 I (can be supercooled to ≈60 °C)			
4-Chloro-1,3-phenylene bis[4,4′-(11-undecenyloxy) benzoyloxy]benzoate (C1Pbis-11BB) Crystal 74 (44 SmC) N 81 I	42	24
		
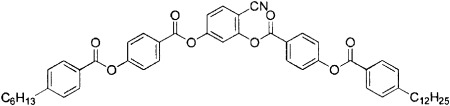	≈60	25
SmX 59 N 122 I (approximate) Also called PAL1 in ref. [25]			
		
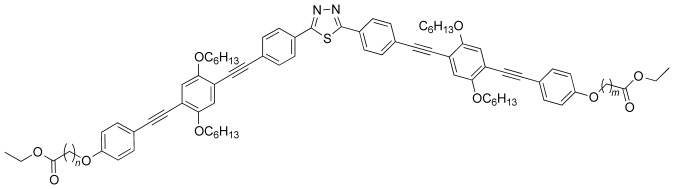	≈140	26, 27
Thiadiazole-based materials. All homologues show broad nematic phases;shorter chains result in lower onset temperatures (two with 4/6 or 4/7 chains lower than 70 °C) and wider phases. Mesophases can be supercooled to room temperature.			
		
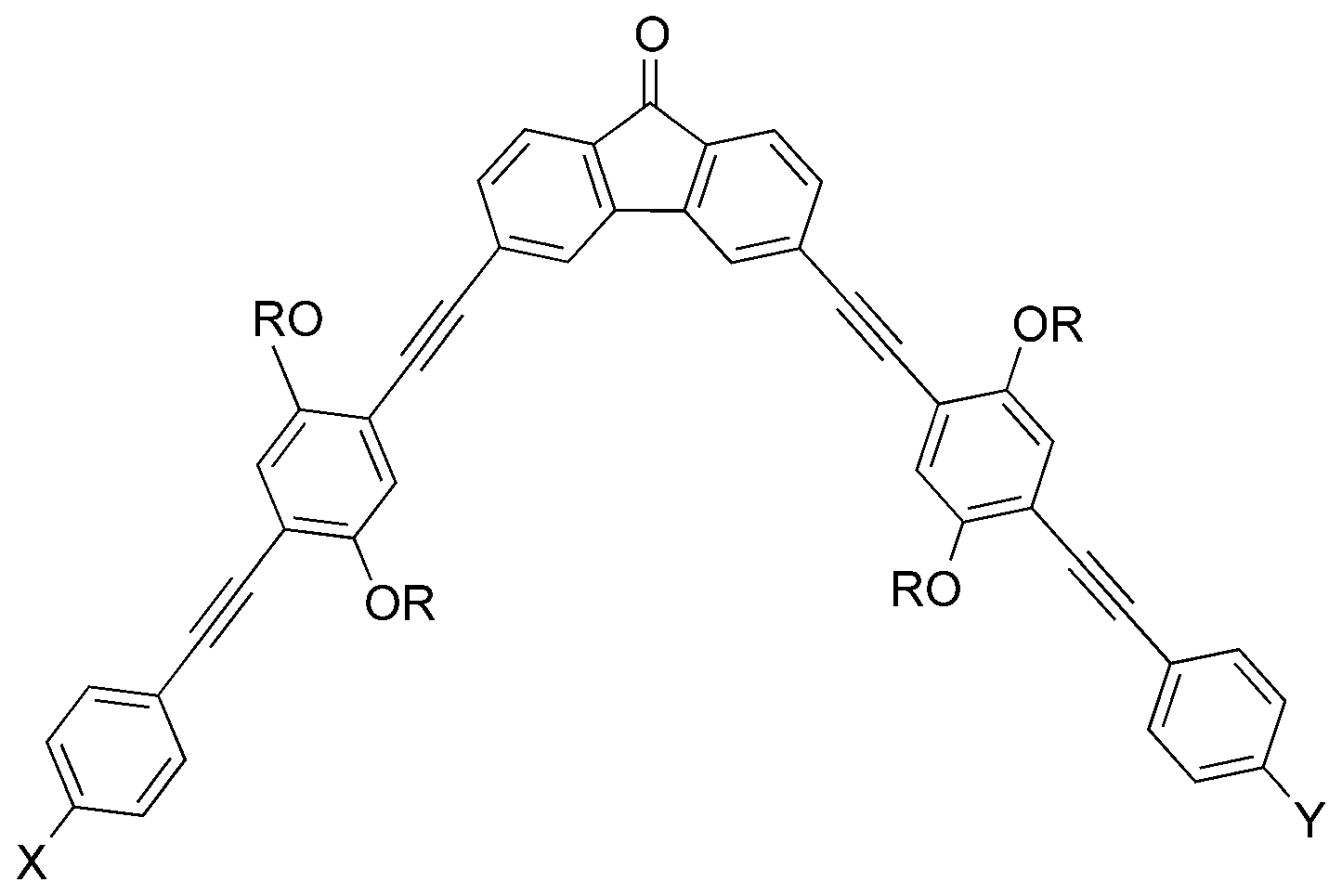	**1 a**–**1 b** ≈145 **1 c** ≈95	28
R=C_6_H_13_ **1 a**: X=H, Y=H. *T*_g_ 29.7 I 104 **1 b**: X=CN, Y=CN. *T*_g_ 34.4 N 178 I **1 c**: X=H, Y=CN. *T*_g_ 26.4 N 121.8 I			

## 3. Physical Properties of the Nematic Phases Formed from Bent-Core Materials

### 3.1. Optical Properties

The high-temperature nematic phase regimes of bent-core mesogens make it relatively difficult to determine their optical properties as approaches such as refractometry cannot be used. Table 6 describes the refractive indices and birefringence of several bent-core materials. The oxadiazole materials (compounds **9**–**12**) have almost indistinguishable values of refractive index and birefringence, thus reflecting the importance of the molecular core in determining the optical properties of nematic liquid crystals. The oxazole material^29^ has apparently reduced values of the refractive indices, but it is to be remembered that the measurement was in this case made at infrared wavelengths, at which dispersion ensures that the refractive indices are lower than at visible wavelengths. Extremely high values of the ordinary and extraordinary refractive indices and the birefringence are reported for a thiadiazole-based material. Again, it is the molecular core that can account for this; as described in ref. 30 there is significant conjugation and there is an absorption edge at wavelengths just below 500 nm. In summary, there are no real surprises with regard to the refractive indices of bent-core nematic liquid crystals; the birefringence is dominated by the molecular core, and in the absence of especially polarisable groups, takes values slightly lower than in cyanobiphenyls as might be expected for molecules with a bent core.

**Table 6:** The ordinary and extraordinary refractive indices, *n*_o_ and *n*_e_, and the birefringence 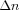 of bent-core nematic materials 10 K below the nematic to isotropic phase transition. Unless indicated otherwise, the optical measurements are made at a wavelength of 589 nm.

Compound	*n*_o_	*n*_e_	Δ*n*	Comments	Refs.
**9**	1.58	1.71	0.13	Δ*n*_max_ ≈0.13	15
**10**	1.59	1.72	0.13	Δ*n*_max_ ≈0.13	15
**11**	1.60	1.73	0.13	Δ*n*_max_ ≈0.16	15
**12**	1.61	1.74	0.13	Δ*n*_max_ ≈0.18	15, 16
**12**-BPO (as in 29)	1.56	1.65	0.09	measured at 1.5 μm	29
thiadiazole	1.57	1.78	0.21	Δ*n*_max_ ≈0.4	30

### 3.2. Dielectric Properties

Dielectric spectroscopy is an important methodology that probes the static and dynamic properties of polar materials, such as liquid crystals, and thus has the potential to distinguish between the subtleties that different kinds of nematic phases may exhibit. Further, the magnitude of the dielectric anisotropy influences the response of the material to an electric field in a device. The oxadiazole materials usually exhibit a small negative dielectric anisotropy (Δ*ε*) across the whole nematic regime, taking values around −3.5, 10 K below the nematic to isotropic transition, and a Freedericksz transition is clearly observed in the homeotropic geometry.^16^, ^17^, ^31^ Early studies of the dielectric behaviour of the oxadiazole nematogens was complicated because the large dipole associated with the core (≈4 D) makes it difficult (but not impossible) to remove ionic impurities from the materials; these can mask important details. A detailed study of Δ*ε* as a function of temperature for compounds **9**–**12**^15^ shows almost identical behaviour of compounds **9** and **11** at the same reduced temperature. The substitution of fluorine on the outer phenyl ring of compound **10** results in the largest value of |Δ*ε*|, which changes from −2.8 at *T*−*T*_NI_=−3.4 K to −4.4 at *T*−*T*_NI_=−14.4 K (*T*_NI_ is the nematic to isotropic phase transition temperature). In compound **12**, Δ*ε* is around 50 % of the value in compounds **9**–**11**, varying from just −1.4 (*T*−*T*_NI_=−8.7 K) to −3.2 (*T*−*T*_NI_=−56.7 K). The dielectric anisotropy of compound **16** is slightly higher than that of compound **9** (see Table 7), which can be attributed to the conjugative mesomeric effect of the phenyl ether oxygen on the lateral fluoro substituent. However, none of the structural variations makes a significant difference to the dielectric anisotropy. The negative dielectric anisotropy and consequent requirement for excellent homeotropic alignment is one of the reasons that the elastic constants have only been determined for rather few materials (see Table 7); the elastic behaviour is discussed in more detail in Section 3.3.

**Table 7:** The dielectric anisotropy 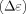 and the splay (*K*_11_), twist (*K*_22_) and bend (*K*_33_) elastic constants (all with units of pN) of compounds 9–13 at a reduced temperature of *T*−*T*_NI_=−10.2 K, in which *T*_NI_ is the nematic to isotropic phase transition temperature for each compound.

Compound	Δ*ε*	*K*_11_	*K*_22_	*K*_33_	Refs.
**9**	−3.1	6.0	0.8	3.7	1, 2, 11, 12, 15 Compound **1** in [15]
**10**	−4.0	6.6	1.2	3.5	12, 15 Compound **2** in 15
**11**	−2.9	6.0	1.1	3.7	12, 15 Compound **3** in 15
**12**	−1.5	3.3	0.6	2.5	12, 15 Compound **4** in 15
**16**	−3.3	5.3	–	3.7	–

**Figure 5:**
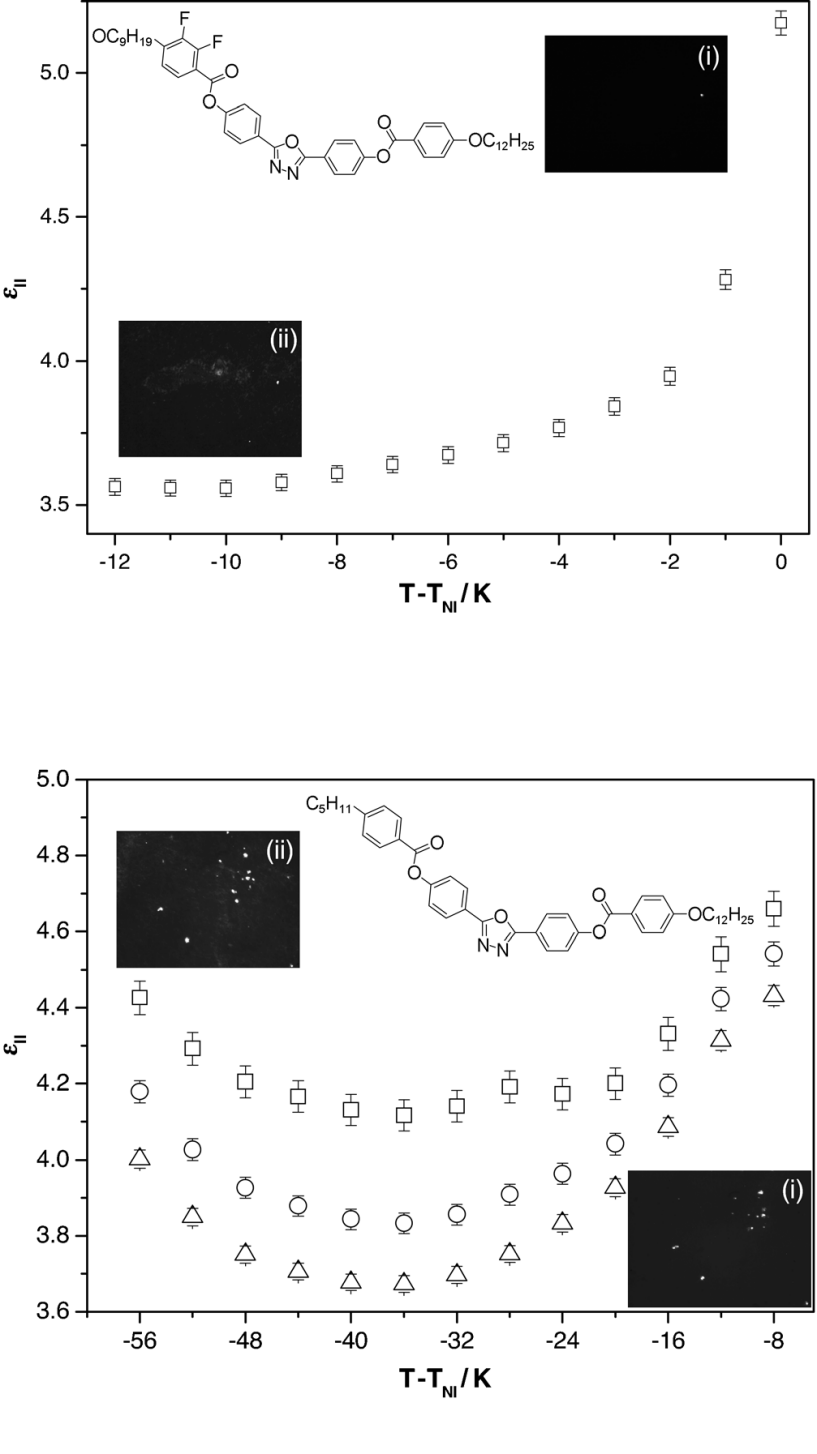
The parallel component of the real part of the dielectric permittivity (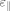) as a function of reduced temperature *T*−*T*_NI_, in which *T*_NI_ is the nematic to isotropic phase transition temperature. (top) Compound 10 in a 3.5 μm thick device (*T*_NI_=210 °C) at 100 kHz. (bottom) Compound 12 in a 5 μm thick device (*T*_NI_=215 °C) at 5, 10 and 100 kHz. The insets show the homeotropic textures observed for compound 10 at *T*−*T*_NI_=−2 and −11 K ((i) and (ii), respectively), and for compound 12 at *T*−*T*_NI_=−10 and −48 K ((i) and (ii), respectively), which indicate uniform alignment as the temperature is reduced.

The dielectric behaviour of compound **10** (Figure 5, top) is typical of the oxadiazole materials when measured in a homeotropic geometry; 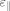
decreases monotonically with decreasing temperature across the nematic regime, as expected. Rather unusual behaviour is observed for compound **12**, however, as shown in Figure 5 (bottom). As the temperature decreases from *T*_NI_, 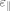 initially decreases as expected for a negative Δ*ε* nematic material in a homeotropic configuration.^32^ However, approximately 35 K below *T*_NI_, 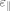 reaches a minimum in compound **12** and thereafter increases with reducing temperature. The insets in Figure 5 show the excellent homeotropic (dark) textures that are observed in the devices for all of the measurements. No change in the alignment is observed that would explain this behaviour.

The anomalous behaviour of compound **12** could, in principle, be explained by multiple relaxations, the appearance of cybotactic clusters, or the emergence of biaxial order. Interestingly though, there is no evidence to support either of the former two possibilities in compound **12**, and there is sparse evidence of biaxiality in any oxadiazole system. Nonetheless, an increase in the dipole–dipole correlation factor due to enhanced alignment of the minor axis in the low-temperature regime, or inclusion of contributions from the biaxial terms in the fundamental expressions for the components of dielectric permittivity^33^ and the emergence of biaxial order would result in such behaviour. This is yet another instance in which unusual behaviour emerges in compound **12** around 35 K below *T*_NI_ (order parameters as measured by Raman scattering,^7^ unusual electroconvection^17^, ^31^ and non-monotonic behaviour of the bend elastic constant^16^), and there is clearly still much to learn about this material.

**Figure 6:**
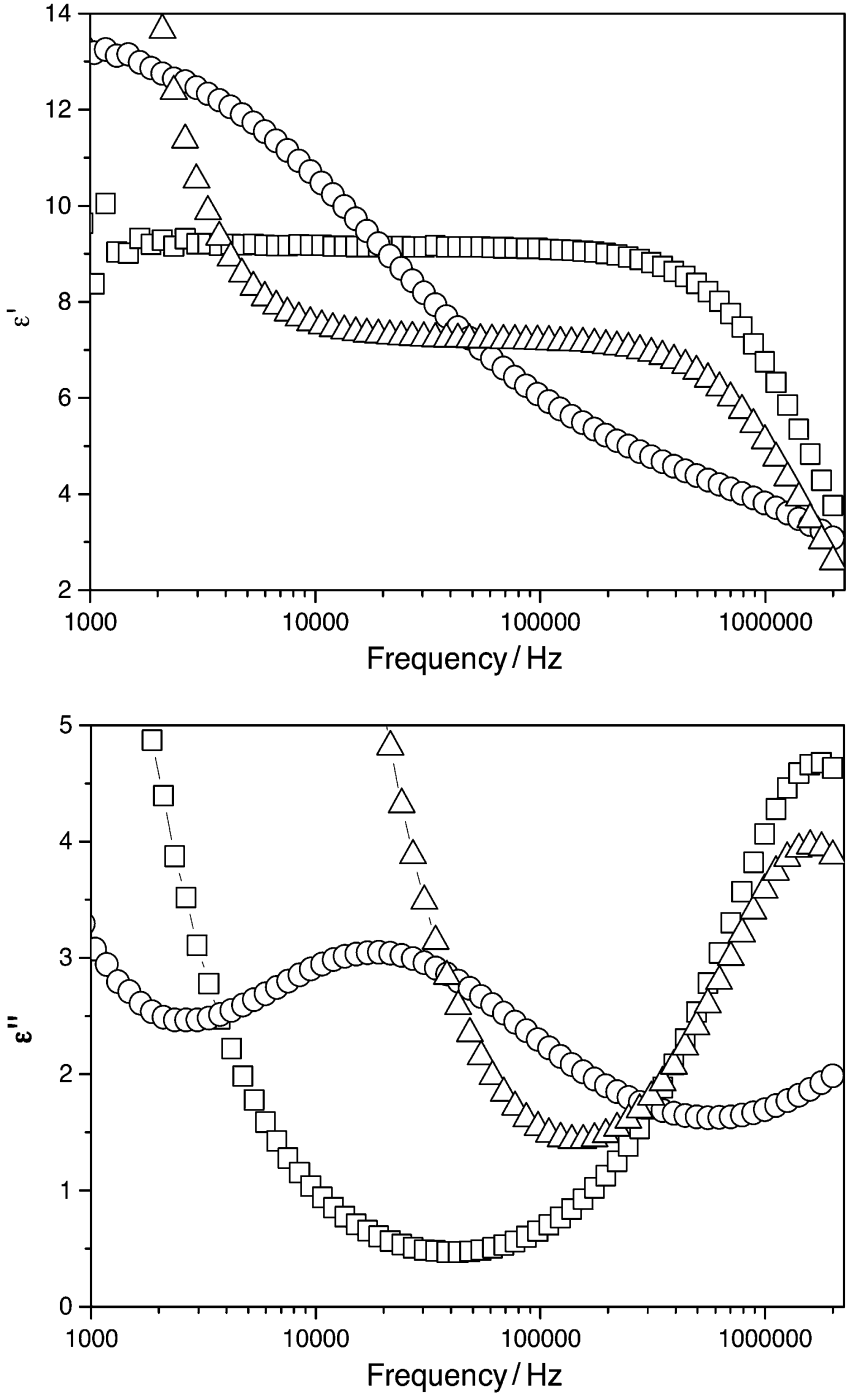
(top) The dielectric permittivity and (bottom) absorption as a function of frequency for compounds 16 (squares), 17 (circles) and 18 (triangles).

As mentioned above, dielectric spectroscopy carried out as a function of temperature can reveal information about the molecular dynamics of a system. Figure 6 a and b show the dielectric permittivity and absorption, respectively, for three different oxadiazole-based nematogens. Compounds **16**–**18** differ in their terminal and lateral groups as shown in Figures 3 and 4. Compounds **16** and **18** are structurally rather different; in particular the outer rings and end groups differ. Compound **16** is similar to compound **9** with respect to the terminal chains but differs in the lateral substitution of MeO and F at S_1_ and S_2_ (see Table 3). Compound **18** exhibits a dielectric permittivity of 7.2 in the region of 10–50 kHz. The effective increase in permittivity observed at frequencies below 1 kHz is due to the relatively high ionic conductivity of this particular material and the interfacial charges that may build up on the indium tin oxide substrates (Maxwell–Wagner effect). On the other hand, compounds **16** and **17** do not show such an increase in permittivity at low frequencies as these materials were specially treated to remove ionic impurities in the materials. The dielectric absorption for both compounds **16** and **17** shows relaxation at approximately 1 MHz, which can be attributed to the rotation of the molecule around its long molecular axis.^34^

**Figure 7:**
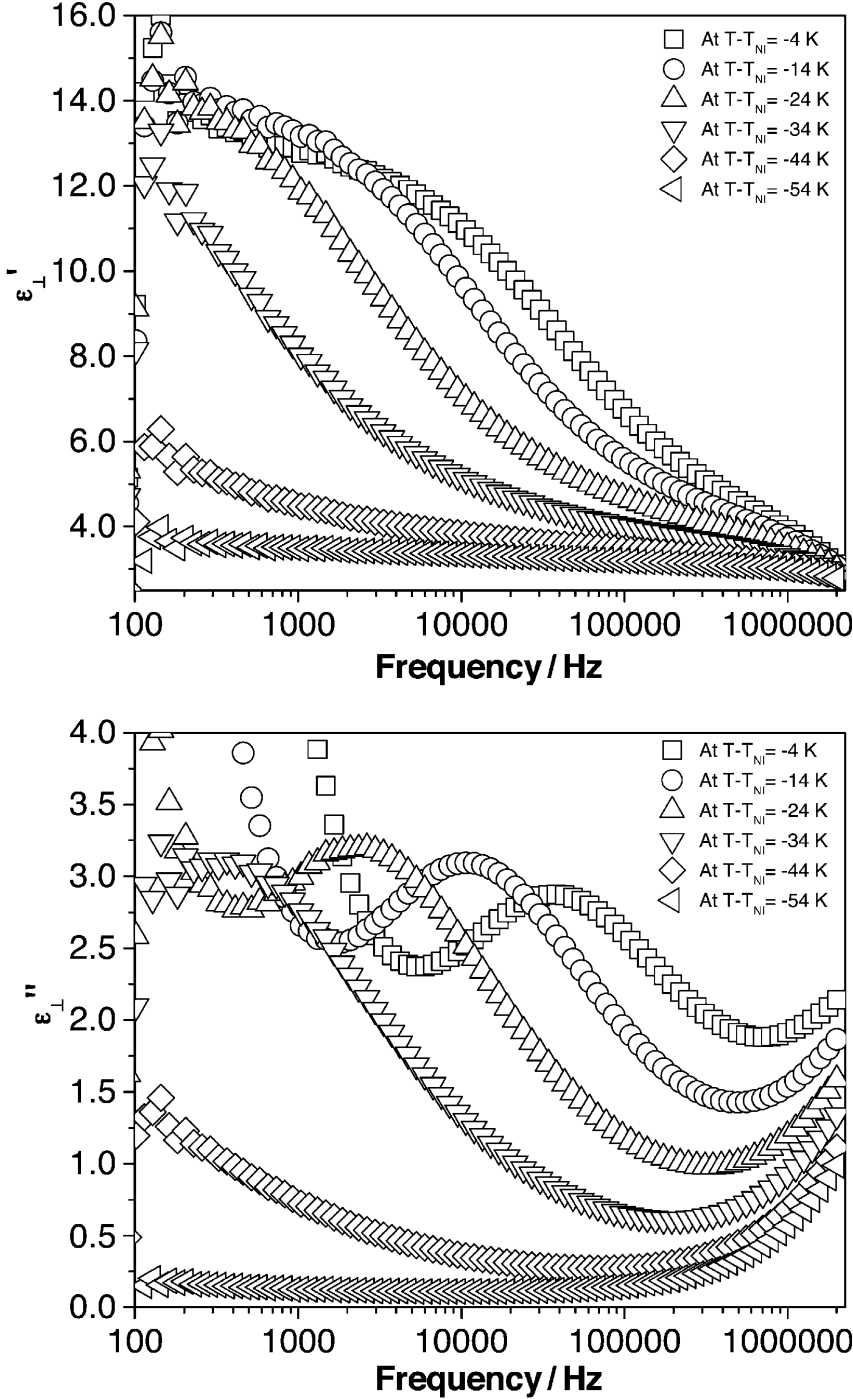
(top) The dielectric permittivity and (bottom) absorption as a function of frequency at different temperatures for compound 17.

It can be seen that the frequency dependence of the dielectric permittivity and absorption of compound **17** is different from that in compounds **16** and **18**. The dielectric permittivity decreases from approximately 13 at 1 kHz to about 6 at 100 kHz with a dielectric relaxation of approximately 20 kHz. Jang et al.^34^ report similar behaviour in a cyanoresorcinol bisbenzoate, though their material exhibits a sign reversal in the dielectric anisotropy at a specific temperature in the nematic regime. In **17** there is no change in the sign of the dielectric anisotropy, or in the planar texture observed at any temperature in the nematic regime (homeotropic alignment was impossible to achieve for this material). The dielectric permittivity for compound **17** mostly decreases with decreasing temperature, though a small increase is observed at low frequencies (<1 kHz) at temperatures above *T*−*T*_NI_=−24 K (Figure 7 a). The dielectric absorption (loss) is found to increase in magnitude with decreasing temperature above *T*−*T*_NI_=−24 K, then decreases (Figure 7 b). The relaxation frequency (as seen from the maximum value of dielectric loss) decreases with decreasing temperature. Such behaviour is similar to that reported by Jang et al.^34^ in the region in which their system exhibited negative dielectric anisotropy. There is no relaxation found for compound **17** at temperatures below *T*−*T*_NI_=−24 K; it appears that the relaxation moves to lower frequencies than those studied in this experiment. Jang et al.^34^ attributed the unusual dielectric behaviour in the resorcinol-based material to cybotactic clusters. Compound **17** has not been studied by X-ray analysis, so it could certainly be the case that such a phenomenon is also responsible here.

The dielectric behaviour of a few other bent-core nematic materials has been studied by various authors. Tadapatri et al.^35^ report the dielectric behaviour of 4-cyanoresorcinol bis[4-(4-*n*-dodecyloxybenzoyloxy)benzoate]; they find that in this system the dielectric anisotropy is again negative, and observe two relaxations in the parallel component and one in the perpendicular component of the dielectric constant. Salamon et al.^36^ describe the behaviour of ClPbis10BB (ref. 23 in Table 5), again observing a negative dielectric anisotropy and more relaxations than are commonly observed in calamitic systems. Indeed, dielectric studies of these complicated nematic systems are both informative and important to deducing other behaviour, such as the elastic coefficients, which is the topic of the following section and it is clear that the dielectric properties of bent-core nematic materials are likely to be of significant interest in the future.

### 3.3. Elastic Properties

The very first measurements of elastic constants in liquid crystals involving bent-core materials were made on mixtures in which the primary constituent was a calamitic nematic material. It was clear in such systems that the bent-core dopant had a strong influence on the bend elastic constant in particular. Measurements of the elastic constants in pure bent-core nematic materials have appeared in the last two to three years, and a characteristic behaviour, such that the bend elastic constant, *K*_33_, is much lower than the splay constant, *K*_11_, has emerged. This trend in the elastic coefficients is the opposite of the behaviour typical for calamitic nematic materials. Most of the measurements of the splay and bend elastic constants reported in the literature are deduced from fits to the Freedericksz transition in homeotropic devices. In-plane devices have been used to deduce the twist elastic constant, *K*_22_, of some materials, though such measurements are inherently less accurate because of inhomogeneous fields. Table 7 summarises the elastic behaviour and dielectric anisotropy of several oxadiazole-based compounds, measured approximately 10 K below the nematic to isotropic phase transition temperature.^15^, ^16^

All of the oxadiazole-based materials studied to date, including those detailed in Table 7, exhibit *K*_22_<*K*_33_<*K*_11_. Further, Majumdar et al.^37^ report the elastic constants at a temperature 2 K below *T*_NI_ in 4-chloro-1,3-phenylene bis-4-[4′-(9-decenyloxy)benzoyloxy]benzoate as *K*_11_=3.1×10^−12^, *K*_22_=0.31×10^−12^ and *K*_33_=0.88×10^−12^ N, deduced by using a combination of Freedericksz transition measurements and dynamic light scattering. Some papers have attributed the low value of *K*_33_ to the existence of cybotactic clusters, and although these clearly occur in many of the bent-core materials studied, they do not appear in all. Indeed it has been shown relatively recently that atomistic calculations that consider the population of conformers in bent-core molecules can reproduce the elastic behaviour of oxadiazoles as a function of temperature with excellent qualitative and quantitative agreement with experiment.^15^, ^16^ It is clear from such calculations that the bend angle is the dominant parameter in determining the elastic behaviour, to such an extent that the “anomalous” finding of *K*_33_<*K*_11_ is associated with the oxadiazole bend angle of approximately 140°, in contrast to *K*_33_>*K*_11_ in the thiadiazole-based materials for which the bend angle is about 164°.^9^, ^30^

It is also worth commenting on the general behaviour of the elastic coefficients in some other bent molecules. Sathyanarayana et al.^38^ report *K*_33_<*K*_11_ for the hockey-stick-shaped compound 4-*n*-butyloxyphenyl [4-(4-*n*-heptyloxybenzoyloxy-4-benzoyloxy)]biphenyl-3-carboxylate, whereas an l-shaped compound^39^ shows similar behaviour. However, a T-shaped compound is reported to exhibit *K*_33_=*K*_11_,^40^ thus indicating that such a system is close to a “cross-over” situation with respect to the conformations that contribute to the elasticity of the material.

The temperature dependence of the elastic constants is also of considerable interest and has been reported by several authors.^15^, ^16^, ^35^, ^41^ It is to be expected that an underlying more ordered phase could have a considerable influence on the temperature dependence of the elastic coefficients, especially the bend constant, close to a smectic phase transition. The compounds studied by Sathyanarayana et al.^41^ and Tadapatri et al.^35^ exhibit a SmC phase immediately below the nematic phase, as do some of the compounds studied by Kaur et al.^15^ All authors describe an approximately linear temperature dependence of *K*_11_, irrespective of the nature of the underlying phase. Kaur et al.^15^ note that the splay elastic coefficient takes values approximately twice as large in oxadiazole compounds with at least one terminal alkoxy chain (compounds **9**, **10** and **11**) as in a compound with a shorter alkyl terminal chain (compound **12**). The strong pre-transitional divergence expected in *K*_33_ for systems with a SmC phase is only clearly observed in one case.^41^ Indeed, *K*_33_ is almost temperature independent in compound **12**, but increases continuously from approximately 1–5 pN in the materials described in ref. 15 and ref. 35. It is perhaps noteworthy that the underlying phase in the case of compound **12** is a dark conglomerate phase, which itself has unusual features.^42^ The unusual temperature insensitivity of *K*_33_ is of significant interest, especially in the context of the unusual phase suggested to be a twist-bend phase, observed in dimer systems that can exhibit elastic behaviour similar to bent-core nematic systems.^43^ Indeed, several years ago, a similar possibility was noted in the nematic phase of some bent-core systems that exhibit an unusual filamentary structure at temperatures close to the transition to a higher-order phase.^13^

### 3.4. Flexoelectric Properties

The flexoelectric behaviour of liquid crystals is of increasing interest as a possible fast electro-optic switching mode for display devices.^44^ It was anticipated from the earliest stages of design of bent-core liquid crystals that their shape could lead to enhanced flexoelectric coefficients. However, as many measurement techniques that deduce information about the flexoelectric coefficients of liquid crystals rely on a detailed knowledge of the elastic constants and the dielectric anisotropy, there are rather few measurements of flexoelectric coefficients currently available for bent-core nematic systems. Salter et al.^45^ find that the flexoelectric difference (*e*_1_−*e*_3_) in the compound 4-cyanoresorcinol bisbenzoate is negative throughout the nematic-phase range and takes values varying from around −10 pCm^−1^ close to the isotropic transition to approximately 17 pCm^−1^ around 10 K below the transition. Kumar et al.^46^ and Van Le et al.^47^ report values of |*e*_3_| of approximately 4 pCm^−1^ (5 K below *T*_NI_) and about 16 pCm^−1^ (10 K below *T*_NI_) for two differently substituted resorcinol derivatives. The temperature dependence of both measurements and calculations of (*e*_1_−*e*_3_) for the oxadiazole-based compound **12** was described recently,^48^ and showed the parameter to take values from −10 to −15 pCm^−1^ across the nematic regime (increasing at lower temperatures). All of these values are somewhat higher than those typically observed in calamitic materials, and all were measured using electro-optic approaches. There is one report of so-called giant flexoelectricity in bent-core nematic compounds,^23^ measured by mechanical flexing, though similar measurements are not found through electro-optic approaches.

## 4. Electric-Field Phenomena

As mentioned above, most bent-core nematic systems have negative dielectric anisotropy and so tend to undergo Freedericksz transitions only in a homeotropic geometry, which is difficult to achieve in such materials. Consequently, many of the electro-optic studies of such bent-core materials have focused on the electroconvection phenomena observed in planar devices. Such studies of compound **12**^17^, ^31^ revealed non-standard electroconvection together with a distinctive change in behaviour around 35 K below *T*_NI_. Indeed, some of the rather beautiful polarising microscopy textures observed are shown in the graphic in the Table of Contents, together with a space-filling model of compound **12**. Non-standard electroconvection has also been reported in 4-chloro-1,3-phenylene bis-4-[4′-(9-decenyloxy)benzoyloxy]benzoate.^49^ Further unusual behaviour has been seen in observations of electroconvection in other bent-core nematic materials, including electrohydrodynamic instability observed above a splay Freedericksz transition in a material with positive dielectric anisotropy.^50^, ^51^

There are very few reports of observations of the much sought-after biaxial switching in the nematic phase of bent-core materials, though Jang et al.^25^ do describe such switching in the material PAL1 (Table 5) with a rather small value (<0.01) for the birefringence associated with the biaxial switching. The unusual dielectric relaxation behaviour that can be seen in bent-core nematic systems, mentioned in Section 3.3, leads us to expect that the electro-optic responses of such systems could be very different from those in calamitic systems. Further, spontaneous chirality in bent-core nematic materials is well documented,^11^–^13^, ^45^, ^52^ and has also been observed recently in hockey-stick-shaped oxadiazole-based materials.^53^ Again, the spontaneous chirality could potentially influence some of the electro-optic behaviour. Indeed, unusual behaviour of reverse twist dislocations in compound **12** under the influence of an electric field has been reported recently.^54^ It has also been reported that electric fields can have a strong influence on the nematic transition temperatures observed in bent-core liquid crystal systems.^55^ The phenomena include a field-induced shift of *T*_NI_ of up to 10 K for fields as low as approximately 1.8 V μm^−1^ as well as the existence of phase transitions associated with cybotactic clusters, driven by the field. It is suggested that the effects are especially significant for systems that have significant cybotactic clusters and no equivalent phenomena are observed in calamitic systems.

## 5. Outlook

Only ten years ago, the excitement surrounding the nematic phases formed from bent-core mesogens was just beginning. Although the highly anticipated thermotropic biaxial nematic phase has proven more elusive than early studies suggested, the nematic phases of these compounds have offered a rich source of new phenomena and effects. Molecular design is now producing systems that exhibit stable nematic phases at relatively low temperatures. Some of the nematic phases are characterised by strong cybotactic clustering. All exhibit elastic behaviour that is quite distinct from that in calamitic systems, and which can be understood in terms of the conformations adopted by the molecules. The flexoelectric behaviour remains of significant interest as it is larger than in calamitic materials. Indeed, the nematic phases formed from bent-core molecules remain fascinating soft matter systems.
